# An eco-friendly evaluation of geraniol and CeO_2_NPs paper poultices for multifunctional paper manuscript conservation

**DOI:** 10.1038/s41598-026-49698-3

**Published:** 2026-05-06

**Authors:** Salwa M. A. Mahmoud, Maisa M. A. Mansour, Maha A. Ali, Mohamed Z. M. Salem

**Affiliations:** 1 Conservation Department, Faculty of Archaeology, Cairo University, Giza, 12613 Egypt; 2 Forestry and Wood Technology Department, Faculty of Agriculture (El- Shatby), Alexandria University, Alexandria, 21545 Egypt

**Keywords:** Paper, Iron Gall Ink, Fungi, Ultraviolet Radiation, Oxidation Geraniol, Ceo_2_NPs, Biological techniques, Chemistry, Environmental sciences, Materials science, Nanoscience and technology, Plant sciences

## Abstract

This study highlights an advanced conservation method for paper manuscripts using eco-friendly methods that are safe, non-toxic, accessible, and feasible. It evaluates geraniol, a plant-derived compound, and cerium oxide nanoparticles (CeO_2_NPs) loaded on Whatman paper poultices. A systematic investigation assessed their individual and combined performance on iron gall ink-treated paper samples. Various analyses were conducted, including USB microscopy, atomic force microscopy, colorimetry, pH measurements, X-ray diffraction analysis, and FTIR spectroscopy analysis. The study aims to determine the most effective approach for treating manuscripts damaged by fungal attacks, specifically *Aspergillus fumigatus* and *Aspergillus terreus*, while minimizing oxidative damage and preserving paper integrity.

## Introduction

 Paper-based culture, such as ancient books, documents, and archives, documents the development of human history, culture, and science^1^. Unfortunate. In humid environments, fungi like *Aspergillus*, *Penicillium*, and *Cladosporium* species produce enzymes, including cellulases, that break down the cellulose in paper^2,3^. These enzymes weaken the paper’s structure, causing physical damage such as embrittlement and loss of mechanical strength. Additionally, fungal growth often appears as visible stains. These stains, which can be red, yellow, brown, or black, are formed by the fungal mycelium, or their metabolism, and from degradation products of the paper^4–6^. Removal of these stains is often challenging without compromising paper integrity. Research indicates a strong correlation between fungal growth on paper and environmental factors like high humidity (60–75% RH) and temperatures (24–28 °C)^7^. Additionally, the presence of nutrients such as gelatin, starch, and other organic materials can exacerbate fungal attacks^8^.

Paper deterioration also results from a series of chemical reactions, such as acidic hydrolysis, alkali degradation, and oxidative degradation, which lead to a reduction in cellulose’s degree of polymerization (DP). On a larger scale, the paper develops a yellowish hue^9^, and the lengthening of cellulose chains into smaller fragments is caused by the oxidation of cellulose. The paper’s tensile strength decreases proportionally with this loss of polymer length, making it more brittle and easier to tear^10^. These oxidative reactions can be accelerated by metallic impurities, such as iron and copper, which are often present in early paper production processes and can speed up the deterioration process^11^.

The conservation of paper artworks requires the use of agents that counteract the effects of fungi and oxidation, preventing further oxidation and stopping fungal growth on paper. These agents work by disrupting fungal cellular metabolism, inhibiting enzyme activity, or damaging the cell Membrane^12^. The aim of using antioxidants and antifungal agents can be achieved by employing nanoparticles and bioactive compounds from plants as multifunctional materials. Nanotechnology is a branch of science that studies particles ranging from one to 100 nanometers in size. These particles, known as nanoparticles, possess unique optical, magnetic, electrical, and mechanical properties that distinguish them from bulk materials. The features of nanomaterials enable various applications^13^. Cerium oxide nanoparticles (CeO_2_NPs) have attracted significant attention as a potential solution for various problems due to their redox activity, ability to scavenge free radicals, and capacity to inhibit biofilm^14,15^. Cerium, a rare earth metal, is the first element in the lanthanide series of the periodic Table^16^.

Cerium exists in nanoparticles in both III and IV oxidation states, providing CeO_2_NPs with excellent antioxidant and catalytic properties^17^. In oxygen-rich environments, cerium ions are primarily Ce (IV), forming stable CeO_2_ structures. In oxygen-poor or reducing conditions, some Ce (IV) ions are converted into Ce (III), leading to oxygen release. The high oxygen storage capacity of cerium oxide mainly comes from the redox cycle between Ce (III) and Ce (IV). Its reversible ability to store and release oxygen ions allows cerium oxide to control the flow and balance of redox reactions^18,19^.

Geraniol (trans-3,7-dimethyl-2,6-octadien-1-ol) is a bioactive, oxygenated monoterpene naturally present in various spices and culinary plants. It is an acyclic alcohol with a floral and slightly fruity-sweet aroma similar to rose^20^, widely used in the flavor and fragrance industry to create and enhance fruity scents such as citrus, raspberry, peach, watermelon, pineapple, or blueberry. Additionally, geraniol can inhibit a broad range of fungi and prevent oxidation^21^.

This study compares the use of CeO_2_NPs and geraniol, both as individual and combined multifunctional compounds, to function as antioxidant and antifungal agents for conserving paper manuscripts with iron gall ink. Iron gall ink was chosen due to its historical use and impact on paper durability. CeO_2_NPs offer advantages over other nanoparticles, including UV protection, compatibility with paper, lower toxicity, and reversible treatments. The treatments’ effectiveness will be evaluated using USB digital microscopy, atomic force microscopy, colorimetry, pH measurements, X-ray diffraction analysis, and Fourier transform infrared spectroscopy analysis.

## Materials and methods

### Paper samples preparation

For the experimental part of this study, 100% cotton paper sheets with a grammage of 25 g/m² were sourced from the Egyptian National Library and Archives in Cairo. The paper sheets were then cut into 50 × 50 mm square samples using a scalpel. The process of making iron gall ink involved mixing the following ingredients: iron(II) sulfate, gum Arabic , tannic acid, and water. Specifically, it required adding 7 g of tannic acid powder to a glass container with 3.3 g of gum Arabic, 14.7 g of iron(II) sulfate, and 100 mL of water. The ink was then applied to one side of the paper using soft, round brushes, with careful attention to prevent contamination^22^.

### Artificial aging and sterilization

The paper samples with iron gall ink underwent artificial aging at 80 °C and 65% relative humidity for 240 h over a period of 10 days, simulating 50 years of natural aging under normal conditions (ISO 5630-3:1996)^23^. Before this process, all samples were sterilized by drying in an oven at 105 °C for 24 h. After treatment, all paper samples were exposed to artificial UV aging by being irradiated with a UV lamp emitting at 350 nm without a filter, placed 25 cm from the sample surface. The samples were exposed to UV light for 120 h at a controlled room temperature of 25 ± 3 °C, according to (ISO-5630-7, 2014)^24^.

### Artificial infection

Spore suspensions (10 mL) of *Aspergillus fumigatus* and *Aspergillus terreus* were isolated from the historical paper manuscript and were prepared, along with a mixed culture of the fungi. The cotton paper and ink paper samples were deliberately inoculated with the fungal species under controlled experimental conditions (27 ± 2 °C and 70 ± 5% relative humidity) for three months^25^.

### Preparation of CeO_2_NPs solution

A dispersion of cerium oxide nanoparticles (CeO_2_NPs) that was purchased from Nano Gate Company with a diameter less than 50 in dimethyl sulfoxide (DMSO) 99.9%, tween 80, and other chemicals used in PDA media were acquired from PioChem Company. was prepared at a concentration of 18.11 µg/mL by mixing 0.90 g of CeO_2_NPs with DMSO to make 50 mL, then sonicated for 2 min using a VCX 500 probe sonicator at the Polymers Laboratory, National Research Centre, Dokki, Egypt.

### Preparation of geraniol solution

Geraniol was purchased from Sigma Aldrich (UK) with a purity 100%. A geraniol solution was prepared at a concentration of 136.7 µL/mL by dissolving 6.83 mL of geraniol in dimethyl sulfoxide (DMSO), with a few drops of Tween 80 as a surfactant. The mixture was sonicated for 2 min to ensure complete dissolution.

### Preparation of Geraniol-CeO_2_NPs mixture

After preparing individual CeO_2_NPs and geraniol solutions, 25 mL of each was mixed and stirred for 5 min.

### Application for treatment agents

To evaluate the efficacy of the treatment agents, experimental papers were immersed in Petri dishes containing treatment solutions. Papers with and without iron gall ink were sandwiched between two sheets of 9 mm Whatman filter paper, saturated with the treatment agents, and secured between two sterile glass slides. After 24 h, the samples were removed from the poultices and allowed to air dry completely. Following treatment, fungal growth on the samples was detected by cotton swabs that were isolated on PDA medium inside Petri dishes, and the isolations were examined every seven days after treatment.

### Treatment of the historical paper manuscript with geraniol poultices

Geraniol, recognized as the most effective treatment agent, was applied to a historical manuscript using a similar method to inhibit fungal growth at a concentration of 136.7 µL/mL.

### USB digital microscope

A USB digital microscope (Model PZ01, Shenzhen Super Eyes Co., Ltd.) with ×1000 magnification was used to examine the samples. The microscope was located in the manuscript laboratory, Department of Conservation, Faculty of Archaeology, Cairo University, Giza, Egypt.

### Scanning electron microscope (SEM) analysis with energy-dispersive X-ray Spectroscopy (EDX)

The surface and structural morphology of the prepared samples were characterized using high-resolution scanning electron microscopy (SEM) on a FEI Quanta FEG 250 instrument at the Desert Research Center in Cairo, Egypt. This advanced field-emission scanning electron microscope, manufactured in the USA, enabled detailed imaging of the sample surfaces. The elemental composition of the materials was determined using energy-dispersive X-ray spectroscopy (EDX), a powerful X-ray technique typically attached to SEM instruments. The EDX analysis was performed on a JEOL JSM-5400LV instrument with an EDX Link ISIS-Oxford attachment at Suhag University’s SEM laboratory, under high-vacuum conditions, with gold coating applied by PSC.

### Atomic force microscope (AFM)

The surface topography of paper samples (2 × 2 cm²) was imaged using an Agilent Technologies 5600LS atomic force microscope (AFM) at the Faculty of Nanotechnology, Egypt. This device delivers high-resolution, three-dimensional data and can resolve features as small as a nuclear lattice for both conductive and non-conductive samples.

### Color change measurements

The color change of the samples was measured with a spectrophotometer (Optimatch 3100, SDL, UK) using a D65 light source and an observation angle of 10°. Measurements were taken in the visible range (400–700 nm) at 10 nm intervals. The color change was quantified using the CIELAB coordinates L*, a*, and b*, which provide a numerical measurement of color differences.

### pH measurements

The pH of the paper samples was measured using a waterproof pH-TEMP pocket tester (AD11, Romania) with a replaceable electrode at Cairo University’s Faculty of Archaeology. The cold extraction procedure was employed, where triplicate samples (1 g) were immersed in 70 mL of cold distilled water (25 ± 5 °C) for 1 h^26^.

### X-Ray diffraction (XRD) analysis

The paper samples’ crystalline structure was examined using an X-ray diffractometer (D2 Phaser 2nd gen, Bruker, Germany) at the Desert Research Center, Cairo, Egypt. The XRD patterns were captured over a 2θ range of 10° to 50°.

### Fourier transform infrared (FTIR) Spectroscopy analysis 

The chemical composition and alterations in the paper samples were analyzed using Fourier transform infrared spectroscopy (FTIR) on a Jasco FTIR spectrometer (Model 6100, Tokyo, Japan) in the project sector, Cairo, Egypt. The spectra were recorded in transmission mode with a triglycine sulfate (TGS) detector, and 2 mm/s coadded scans across the spectral range 4000 –400 cm⁻¹, with a resolution of 4 cm⁻¹ by the ATR-FTIR method.

## Results

### Fungal growth measurement

**Fig. 1:**
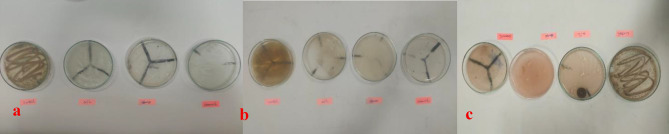
The fungal growth after (**a**) 35, (**b**) 54, and (**c**) 61 days compared to the control sample.

Cotton swab samples were taken from the treated sample with geraniol, CeO_2_NPs, and a combination of both agents, which were inoculated and monitored for fungal growth. The results indicated no observable fungal growth in the treated samples over a continuous 30-day period. However, after 35 days and subsequently 54 days, fungal growth began to clear on the sample treated with geraniol alone (Fig. 1). In contrast, the CeO_2_NPs treatment demonstrated inhibition of fungal growth for *Aspergillus terreus* and *Aspergillus fumigatus*, persisting for 61 days, which exhibited the longest duration of antifungal efficacy on the treated samples exposed to uncontrolled environmental conditions.

### USB Digital Microscope

**Fig. 2:**
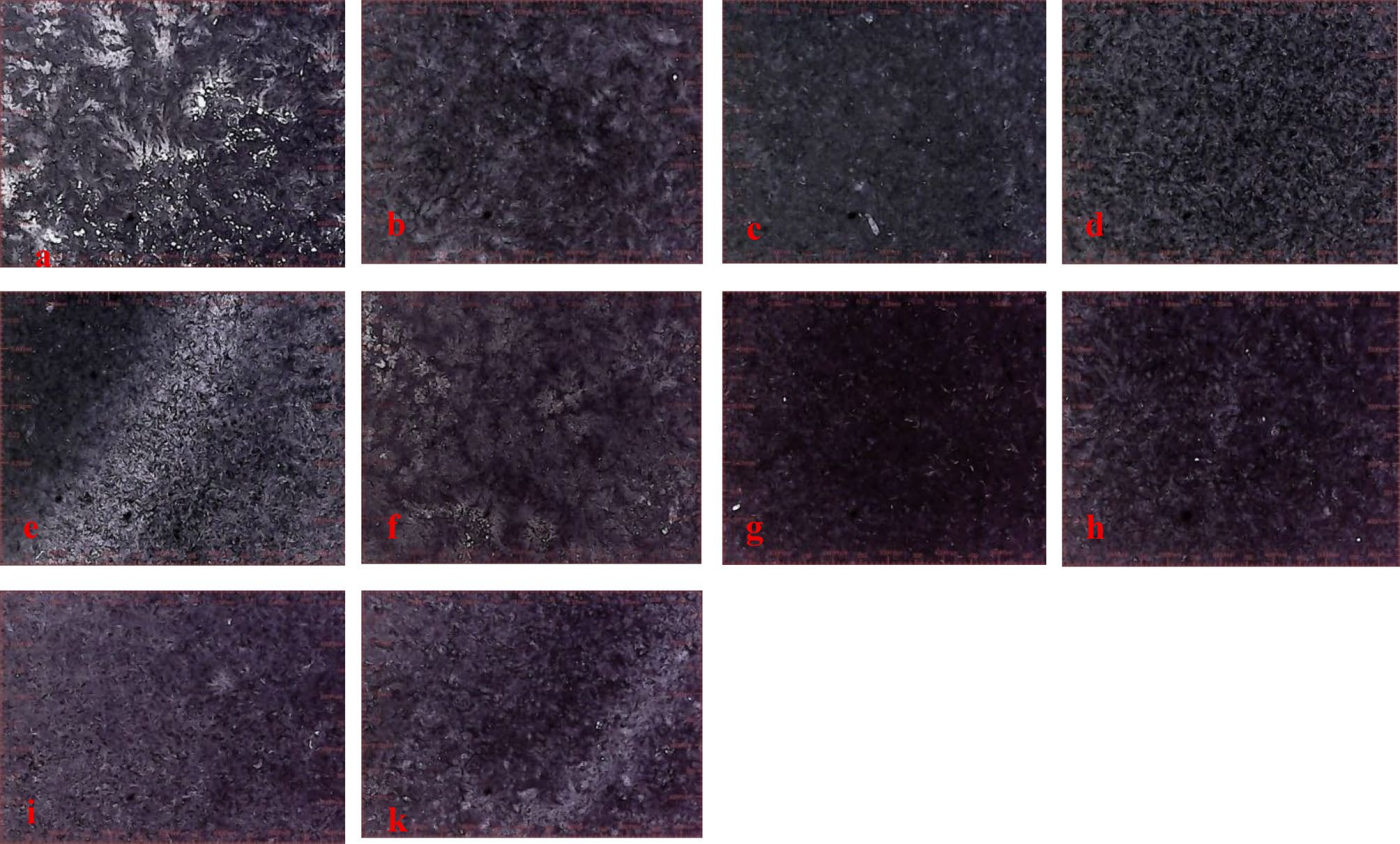
(**a**) Control sample with iron gall ink salts appearing white, (**b**) the sample after fungal infection with a darker appearance compared to the untreated sample before aging, (**c**) the sample treated with geraniol before aging, (**d**) the sample treated with CeO_2_NPs before aging, and (**e**) the sample treated with a mixture of both materials before artificial aging. Additionally, (**f**) aged untreated sample with cracks in the iron gall ink layer, (**g**) the infected sample after fungal infection after aging, (**h**) the sample treated with geraniol after aging, (**i**) the sample treated with CeO_2_NPs, and (**k**) the sample treated with a mixture of both materials before aging and after artificial aging.

The digital microscope was utilized to image the surface and detect the impact of various aging processes on the iron gall ink. The results revealed that untreated samples exposed to thermal and humidity aging exhibited salt formation on their surface, resulting in a color change from black to gray. Upon application of the fungal suspension, redissolution of some salts on the surface was observed. However, treatment with geraniol restored the iron ink’s black color without any damage. Similarly, treatment with CeO_2_NPs yielded comparable results with a slight fading compared to geraniol. In contrast, treatment with a mixture of geraniol and CeO_2_NPs resulted in a noticeable color alteration and significant fading of the ink layer. Following exposure to artificial aging via UV radiation, the aged untreated sample exhibited increased cracking and fragility of the ink layer. Conversely, geraniol-treated samples exhibited minimal iron ink color change post-aging. In contrast, CeO_2_NPs or geraniol-CeO_2_NPs-treated samples showed noticeable iron ink fading versus aged untreated samples under UV aging (Fig. 2).

**Fig. 3:**
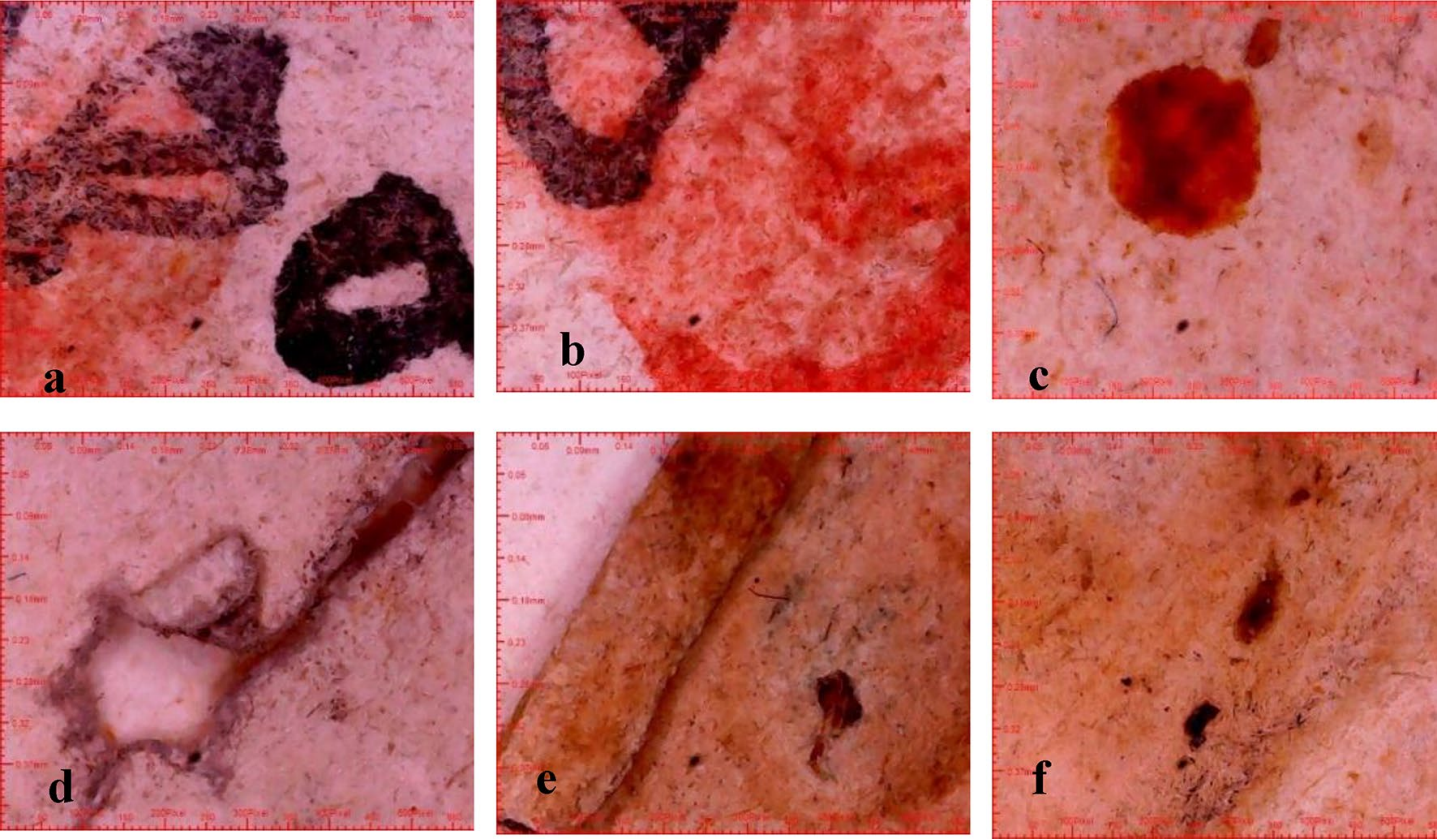
The deterioration of the manuscript, including (**a**) flaking of carbon ink, (**b**) vermilion ink, (**c**) along with stains, (**d**) holes, (**e**) flexure, and (**f**) evidence of fungal growth and insect damage.

The paper manuscript from the Ottoman era has been subjected to a multitude of degradation factors, resulting in a range of detrimental effects on its physical and aesthetic integrity. These include chromatic alterations and uneven discoloration of the carbon ink and vermilion ink, as well as the presence of dark brown insect residues and particulate deposits dispersed across the surface. Furthermore, the manuscript exhibits extensive losses in some parts, likely exacerbated by inadequate storage conditions that have induced creasing and folding along the page edges. The cumulative impact of these factors, compounded by the potential for fungal growth and moisture-induced degradation, has likely contributed to the manuscript’s current state of fragility and susceptibility to further deterioration (Fig. 3).

### Scanning electron microscope with EDX analysis

**Fig. 4:**
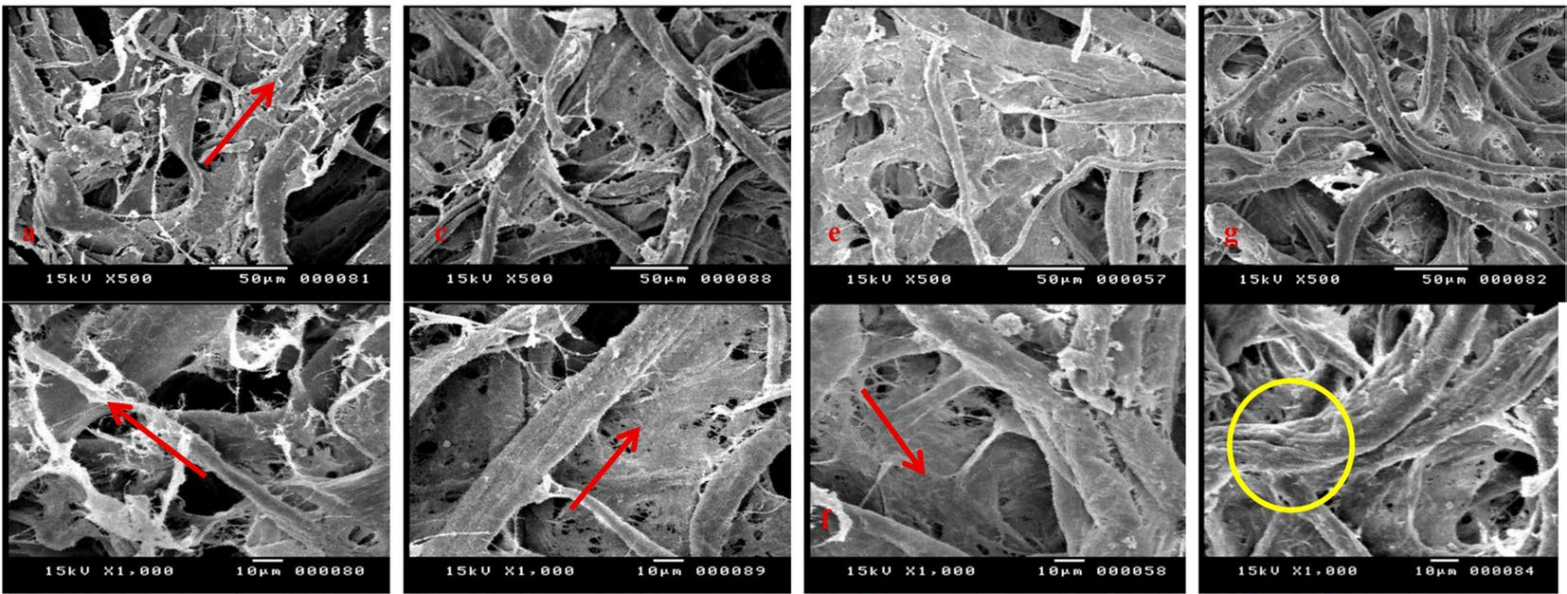
(**a**,** b**) The control paper samples before and after aging, (**c**,** d**) the sample treated with geraniol before and after aging, (**e**,** f**) the sample treated with CeO_2_NPs before and after aging, and (**f**,** h**) the sample treated with a mixture of both materials before and after aging.

Scanning electron microscopy (SEM) imaging offers detailed insights into the physical features of fibers and the degree of damage caused by fungal attack, clarifying the effects on fiber structure. The SEM images of untreated paper samples show the characteristic shape of cotton fibers, along with signs of deterioration from accelerated aging involving heat and humidity, which lead to fiber damage and gaps. After artificial inoculation with *Aspergillus fumigatus* and *Aspergillus terreus*, there is clear evidence of fungal hyphae wrapping around the cotton fibers, along with an increase in gaps and breakdown and erosion of the fibers. In contrast, SEM images of the samples treated with geraniol before fungal inoculation reveal the absence of fungal hyphae and conidiophores. Instead, the fibers form a tight network, with geraniol coating the fibers and maintaining their structure. Even after aging, the fibers stay close together, and the network remains intact, protecting against the damaging effects of aging (Fig. 4).

**Fig. 5:**
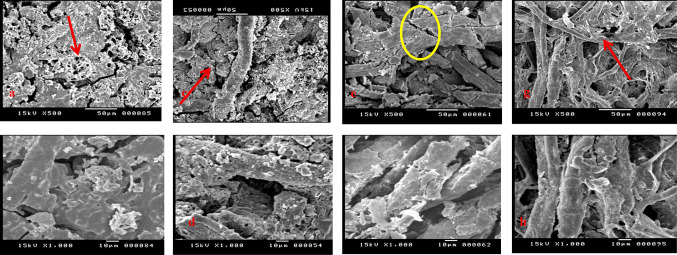
The paper samples with iron gall ink pre- and post-aging: (**a-b**) control, (**c-d**) geraniol-treated, (**e-f**) CeO_2_NPs-treated, and (**g-h**) treated with both, showcasing treatment effects.

In the context of paper samples coated with iron gall ink, it is noteworthy that the sample subjected to artificial aging through heat and humidity exhibits pronounced salt efflorescence on the fiber surface. This phenomenon results from heat’s dehydrating effect, which causes evaporation of water within both the fibers and the ink, leading to the precipitation of its components onto the surface. When the samples are inoculated with fungi, a noticeable presence of salts on the surface is observed, although less than the efflorescence seen in the aged, untreated samples. This reduction may be due to the fungal suspension, containing deionized water, redissolving and redistributing the salts on the surface with the ink. After treating the ink with geraniol, a clear, uniform layer forms on its surface, effectively coating it. Subsequent exposure to artificial aging with UV radiation shows that the fibers remain coated with geraniol, maintaining their structure without significant effects (Fig. 5).

**Fig. 6:**
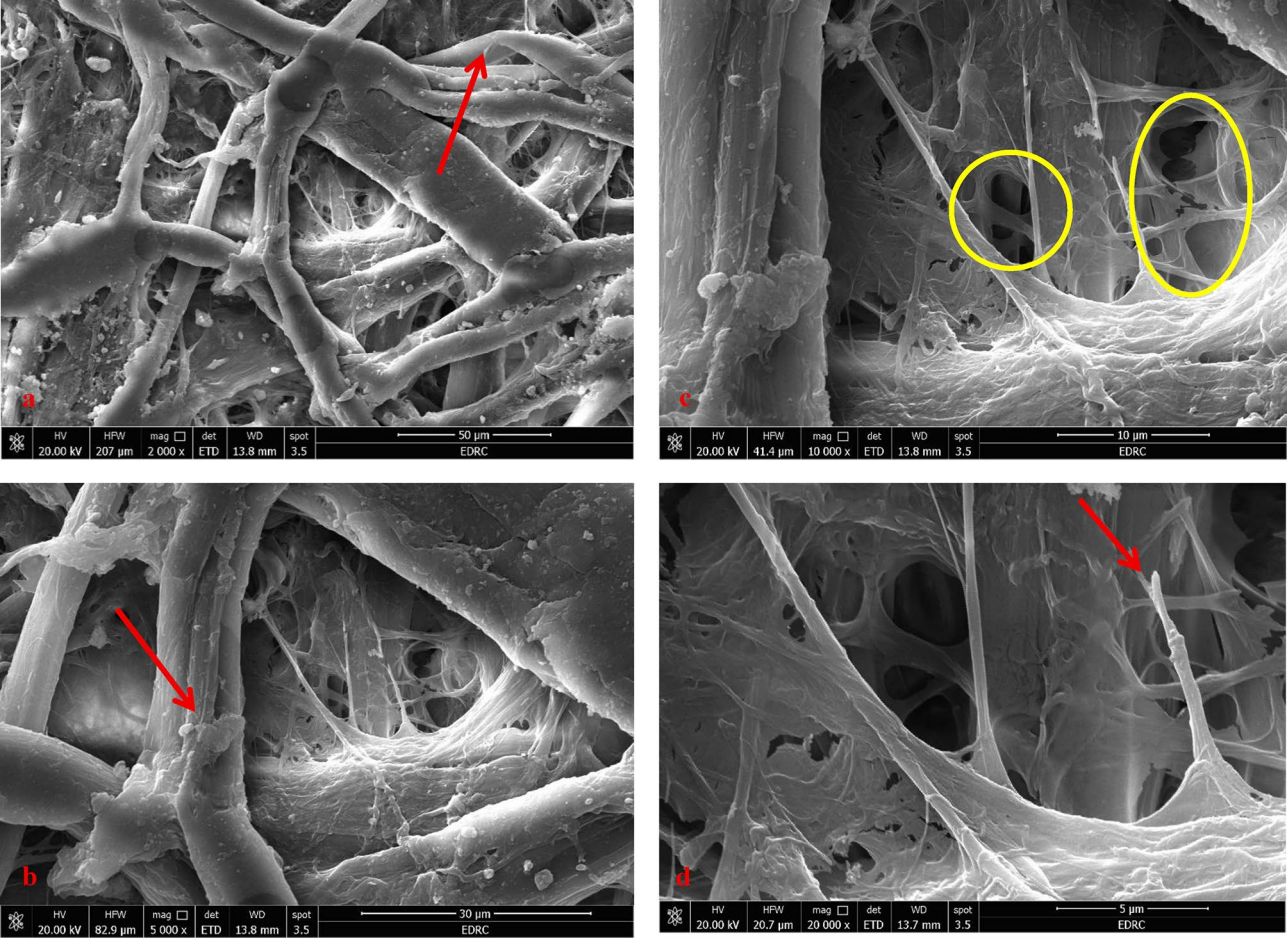
(**a**) The cotton fibers of the paper manuscript, (**b**) characterized by tears, (**c**) gaps, and (**d**) erosion of fibers, providing insight into the degradation of the paper.

**Fig. 7:**
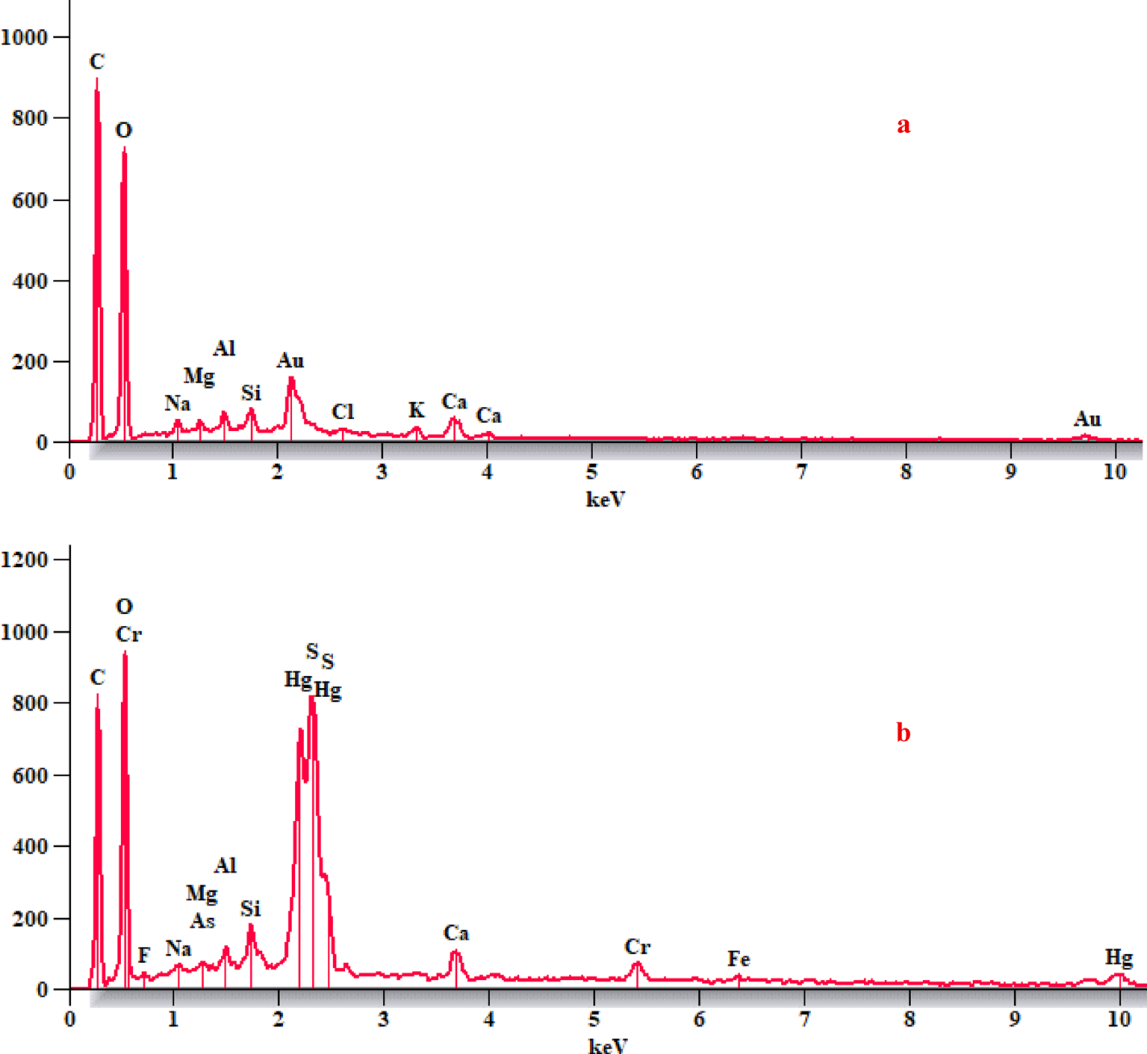
EDX analysis of (**a**) carbon ink, and (**b**) vermilion red.

A thorough examination of the paper sample from the paper manuscript shows it is made of cotton fibers that have suffered significant degradation from fungal infection. This microbial damage has led to notable fiber deterioration, including delamination and fragmentation, as well as widespread spread of fungal spores on the fiber surfaces and an increase in gaps. Additionally, the cell walls of the cotton fibers show clear signs of weakness and tearing, a common feature in paper affected by fungal infection. Using energy-dispersive X-ray spectroscopy (EDX) with scanning electron microscopy (SEM), a detailed analysis of the black ink was performed, which clearly identified carbon as the main element in the black ink. This strongly suggests that the ink is carbon ink. On the other hand, a careful analysis of red ink found sulfur and mercury, which are the main elements in vermilion red (mercuric sulfide), providing definitive proof of the ink used (Figs. 6 and 7).

### Atomic force microscope

**Fig. 8:**
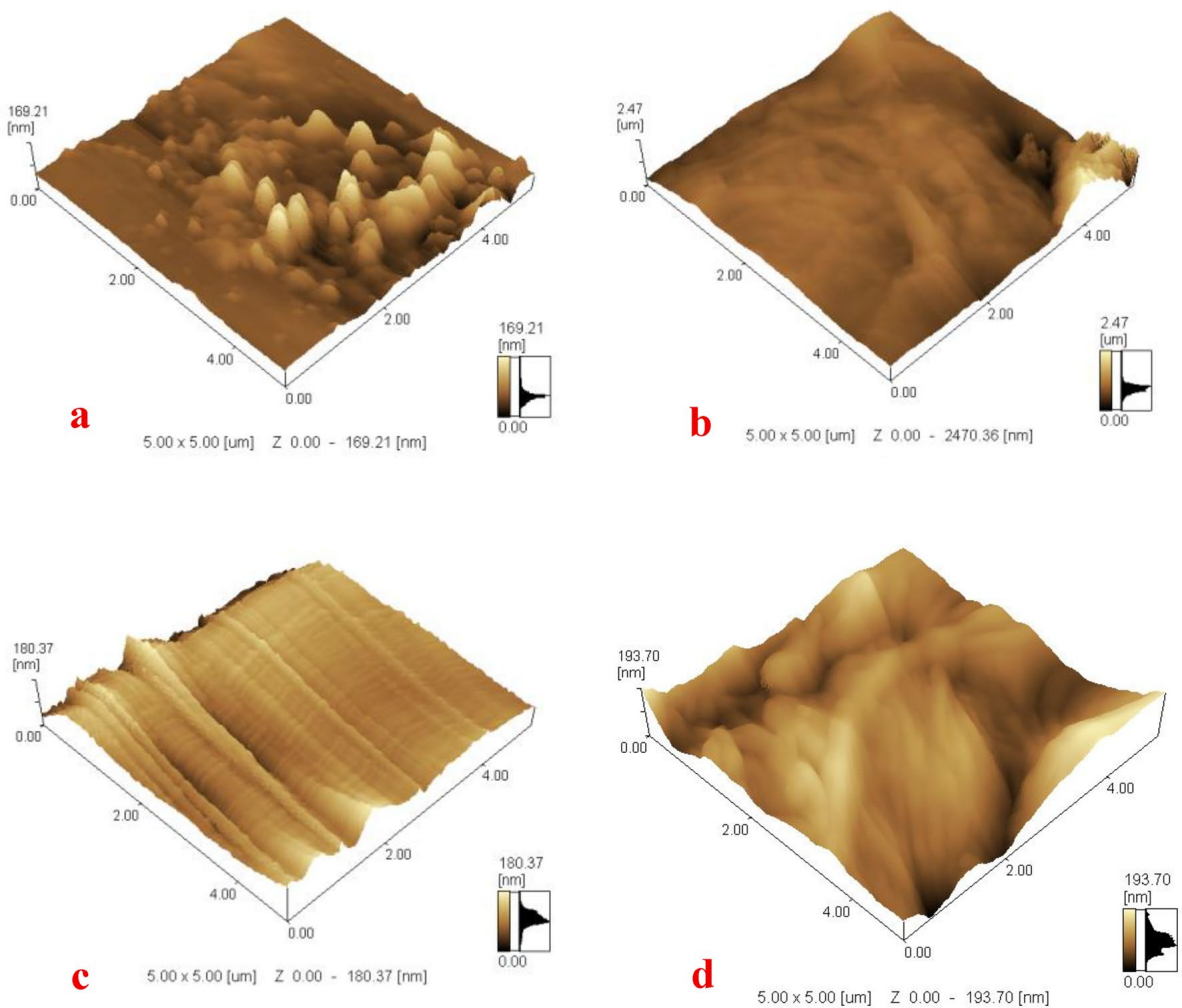
(**a**) The control paper sample, (**b**) the infected paper sample, (**c**) the treated sample with geraniol before aging, and (**d**) the treated sample with geraniol after aging.

Atomic force microscope (AFM) imaging shows that the untreated sample has a surface roughness of 169.21 nm. After artificial inoculation with a fungal suspension containing *Aspergillus fumigatus* and *Aspergillus terreus*, there is a significant increase in surface roughness, reaching 2470.36 nm. This major increase is due to fungal colonization on the paper surface and the release of metabolites and acids, which work together to worsen surface degradation and roughness. In contrast, paper samples treated with geraniol using the paper poultice method with geraniol display a surface roughness of 180.37 nm, similar to that of the untreated sample before fungal inoculation. This suggests that the treatment effectively cleans the fungal growth and prevents surface damage. Additionally, after exposure to accelerated aging by UV, the treated samples show minimal changes, with a surface roughness of 193.70 nm, indicating that the surface remains relatively stable and resistant to degradation even after artificial aging (Fig. 8).

### Color change measurements

Color change measurement is an essential tool for detecting changes caused by damage or treatment with different materials, as well as evaluating the effects on paper or iron ink samples after UV-induced aging. A comparison of the sample infected with the fungal suspension showed a color change of 2.18 compared to the untreated sample, characterized by increased yellowing, evidenced by the b* value rising from 2 in the standard sample to 3.81 in the infected sample. Treatment with various materials resulted in different color change values, including 3.35 for CeO_2_NPs, 3.24 for the sample treated with geraniol and CeO_2_NPs, and 3.97 for the sample treated with geraniol. Notably, the b* value increased significantly compared to changes in L* and a* values relative to the control sample before artificial aging. After exposure to UV aging, the color changes were minimal in the treated samples. Specifically, geraniol caused a negligible color change of 1.94, while CeO_2_NPs resulted in a change of 1.81. In contrast, the mixture of both materials produced a color change of 1.05.

Notably, the most pronounced color change occurred in the ink sample infected with fungal suspension, with a value of 12.49. This significant alteration was attributed to the fungal infection and the resulting fungal secretions, which led to a marked decrease in the L* value from 23.32 to 17.63, indicating a noticeable darkening of the infected sample compared to the standard sample containing iron ink. Conversely, the lowest color change rates among the treated samples were observed for geraniol, which induced a color change of 1.79, and the mixture of geraniol with CeO_2_NPs, which resulted in a color change of 0.56. In contrast, treatment with CeO_2_NPs alone yielded a slight color change of 3.5 compared to the other treatment materials. Furthermore, it is observed that the magnitude of color change in samples containing iron ink after exposure to UV aging ranged from 0.73 to 2.09, compared to the samples treated before aging. This indicates that the samples exhibited minimal impact from UV exposure, suggesting a relative stability of the treated samples to UV aging (Table 1).

**Table 1:** The colorimetric changes in the paper samples and iron gall ink samples were treated with geraniol, CeO_2_NPs, and a geraniol-CeO_2_NPs mixture, both before and after artificial aging.

Samples	(L)	(a)	(b)	∆E
Before aging				
Control paper sample	90.74	0.45	2.00	-
Control ink sample	23.32	-6.74	9.54	-
Infected paper sample	89.55	0.70	3.81	2.18
Infected ink sample	17.63	0.38	1.00	12.49
Treated paper sample with geraniol	90.26	1.11	7.70	3.97
Treated ink sample with geraniol	18.35	-1.26	0.91	1.79
Treated paper sample with CeO_2_NPs	90.59	1.06	6.98	3.35
Treated ink sample with CeO_2_NPs	18.58	-2.72	2.43	3.5
Treated paper sample with geraniol and CeO_2_NPs	90.24	1.26	7.12	3.42
Treated ink sample with geraniol and CeO_2_NPs	18.15	0.25	0.82	0.56
After aging				
Control paper sample	90.22	0.78	4.27	2.35
Control ink sample	24.31	-6.25	9.25	1.14
Infected paper sample	89.70	0.92	4.76	0.72
Infected ink sample	18.50	0.56	1.38	0.96
Treated paper sample with geraniol	91.24	0.92	5.95	1.94
Treated ink sample with geraniol	18.13	0.29	0.80	0.73
Treated paper sample with CeO_2_NPs	91.45	0.73	5.21	1.81
Treated ink sample with CeO_2_NPs	17.71	0.12	0.78	1.08
Treated paper sample with geraniol and CeO_2_NPs	90.56	0.88	5.37	1.05
Treated ink sample with geraniol and CeO_2_NPs	18.73	-1.37	2.17	2.09

### pH measurements

The measurement of pH value is a key analytical parameter that reveals the effects of different treatments on paper, as well as the extent of deterioration caused by fungal growth and its impact on the paper’s pH. The control paper sample had a pH of 5.6 due to exposure to accelerated aging caused by heat and humidity. After artificial fungal infection, a drop in pH was noted, reaching 5.2. However, when treated with geraniol and CeO_2_NPs, the pH remained fairly stable. In contrast, using both substances together caused a notable increase in pH from 5.2 to 5.5. Importantly, paper samples treated with various substances showed excellent stability and resistance to degradation after accelerated aging with UV radiation, with no major changes in their physical or chemical properties.

**Fig. 9:**
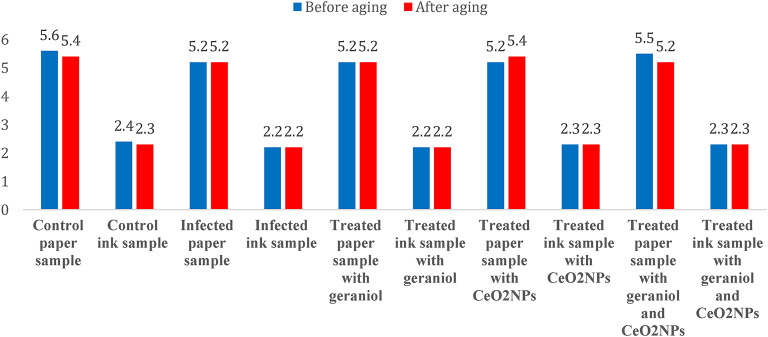
the pH measurements in the paper samples and iron gall ink samples were treated with geraniol, CeO_2_NPs, and a geraniol-CeO_2_NPs mixture, both before and after artificial aging.

Furthermore, in the paper samples with iron gall ink, the pH was measured at 2.4, indicating an acidic condition caused by the ink. After treatment with geraniol, the pH stayed close to that of the infected sample at 2.2. Similarly, treatment with CeO_2_NPs alone or combined with geraniol yielded pH values of 2.3 both before and after accelerated aging with UV radiation. This shows that the treatments did not significantly alter the acidic pH of the paper samples containing iron gall ink (Fig. 9).

### X-Ray diffraction analysis

**Fig. 10:**
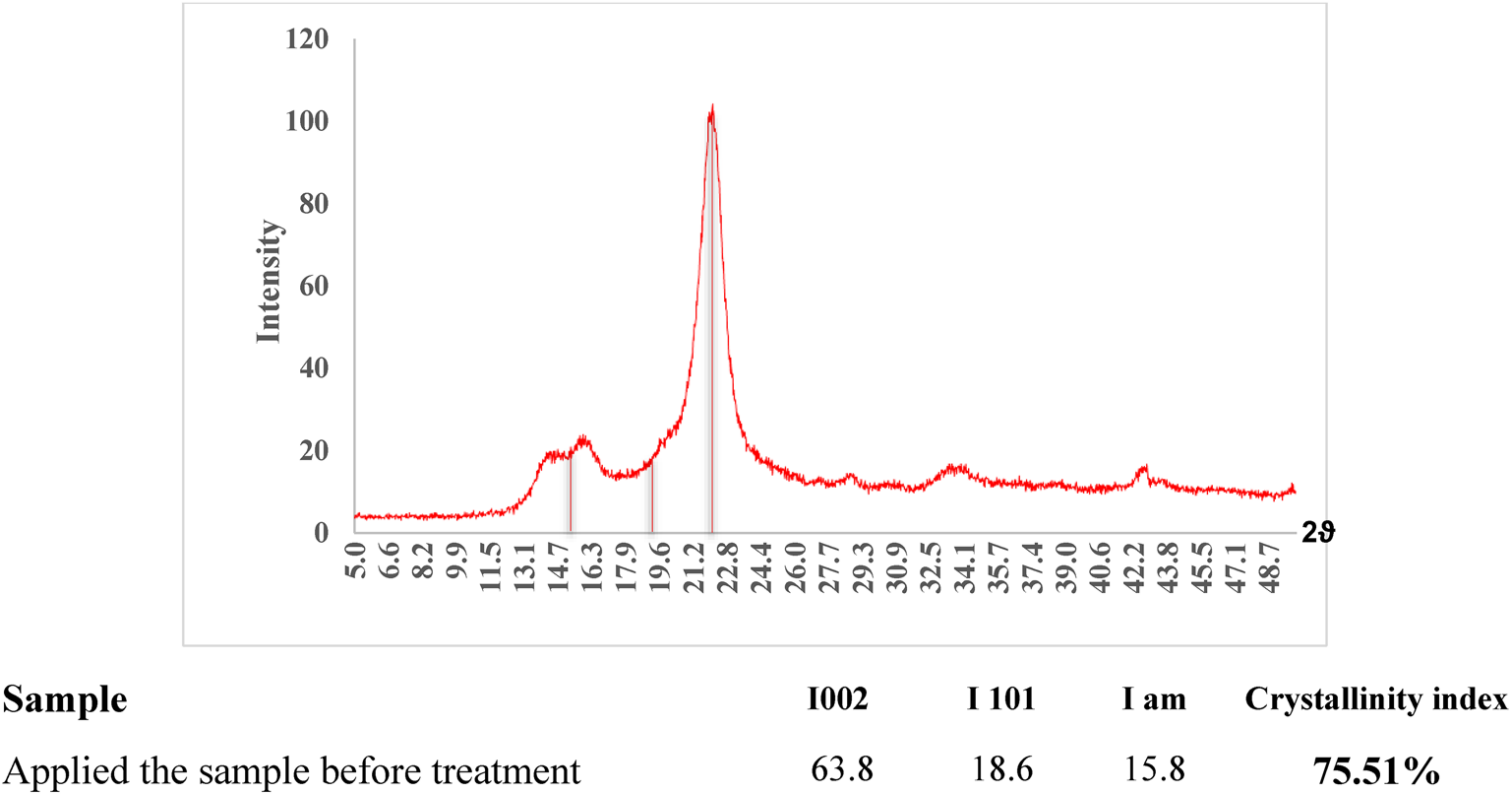
XRD analysis of the paper sample from the paper manuscript before treatment.

The X-ray diffraction analysis of the paper manuscript showed exposure to various degradation factors, which caused damage to the cellulose structure. This led to a decrease in the crystallinity of cellulose to 75.51%, falling below the normal range. This indicates that the fibers have undergone significant deterioration, including the breaking of cellulose fibers, resulting in lower crystallinity. It also reflects the manuscript’s fragility and vulnerability. Therefore, this value emphasizes the need for consolidation treatments to boost cellulose crystallinity and protect the paper from further damage according to Segal’s Eq.1 (Fig. 10).

$$\:Equ1:\:Cri=\frac{I002-Iam}{I002}\times\:100$$

### Fourier transform infrared spectroscopy analysis

**Fig. 11:**
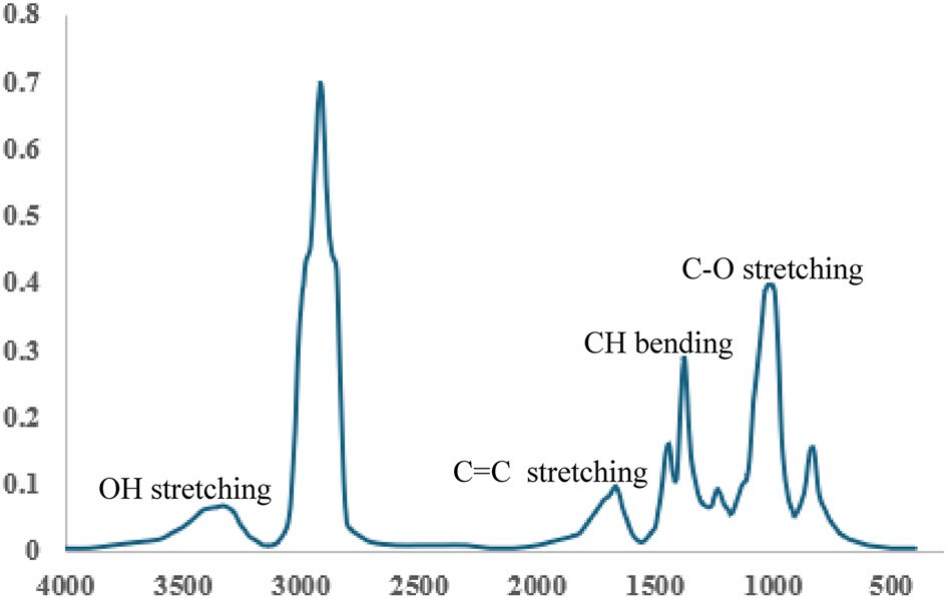
FTIR analysis of geraniol.

**Fig. 12:**
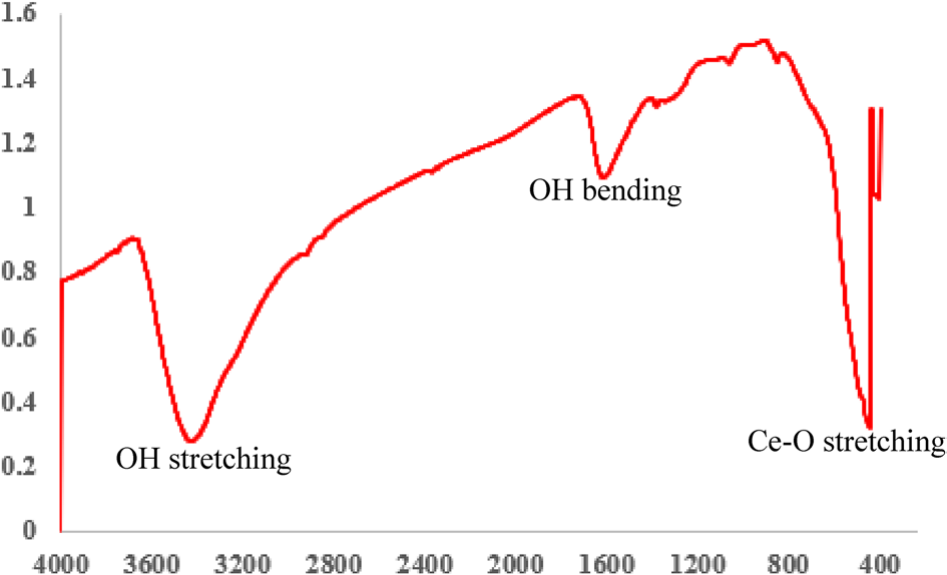
FTIR analysis of CeO_2_NPs.

Geraniol is an organic compound with the chemical formula C_10_H_18_O. The characteristic functional groups of geraniol include a hydroxyl group (-OH) that appears at a specific wavenumber at 3332 cm^− 1^, an alkene group (C = C stretching) that appears at 1668 cm^− 1^, a methyl group (-CH_3_)(C-H bending) that appears at another wavenumber 1378 cm^− 1^, a methylene group (-CH_2_-)(C-H bending) at 1445 cm^− 1^, and a C-O stretching vibration that appears at 1020 cm^− 1^(Fig. 11), and the FTIR groups of CeO_2_NPs typically include O-H stretching appears at 3457 cm^− 1^, O-H bending appears at 1693 cm^− 1^, and Ce-O stretching appears at 433 cm^− 1^(Fig. 12).

The infrared spectroscopy analysis of the paper samples reveals no significant changes in their functional groups after treatment with geraniol, CeO_2_NPs, or their combination. This suggests that these substances did not induce notable alterations in the functional groups of the paper, except for a minor shift in the wavelength of the OH bending band from 1642 cm⁻¹ to 1630 cm⁻¹ when treated with geraniol.

Regarding the samples containing iron gall ink and treated with various materials, it is observed that the aged untreated sample under heat and humidity exhibited a wavelength value of 3232 cm^− 1^ for OH stretching and a C = O stretching band around 1701 cm^− 1^. The degree of crystallinity reached 1441 cm^− 1^. After exposure to artificial aging through fungal infection, a significant decrease was noted from 3232 cm^− 1^ in the control sample to 3177 cm^− 1^ in the infected sample. Additionally, the band associated with the C = O region disappeared, and crystallinity decreased from 1441 cm^− 1^ to 1433 cm^− 1^.

However, treatment with geraniol before and after exposure to aging showed no presence of oxidation bands, indicating that the treatment protected the paper and its iron gall ink content from oxidation effects, with the oxidation band disappearing entirely. In contrast, treatment with CeO_2_NPs resulted in an OH stretching wavelength of 3339 cm^− 1^ before aging, which decreased to 3267 cm^− 1^ after UV aging. Furthermore, a new band in the C = O region appeared at 1706 cm^− 1^ after aging but was absent before treatment.

**Fig. 13:**
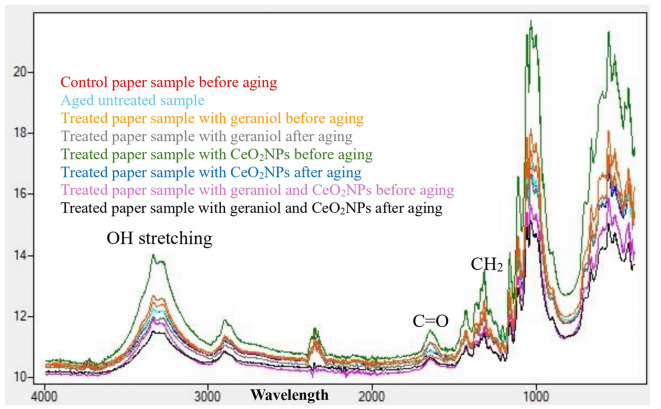
The control paper sample, the treated paper samples with geraniol, CeO_2_NPs, and a geraniol-CeO_2_NPs mixture, both before and after artificial aging.

Treatment with a mixture of both materials showed no significant change in OH stretching, with values of 3192 cm^− 1^ before treatment and 3191 cm^− 1^ after treatment. However, after UV aging, a band associated with C = O stretching appeared at 1702 cm^− 1^, which was not present before treatment. This indicates that the mixture of materials was affected by UV aging processes. These findings suggest that geraniol is the most effective material when used at a concentration of 13*6.7* µL/mL, applied alone without mixing with CeO_2_NPs (Figs. 13 and 13).

**Fig. 14:**
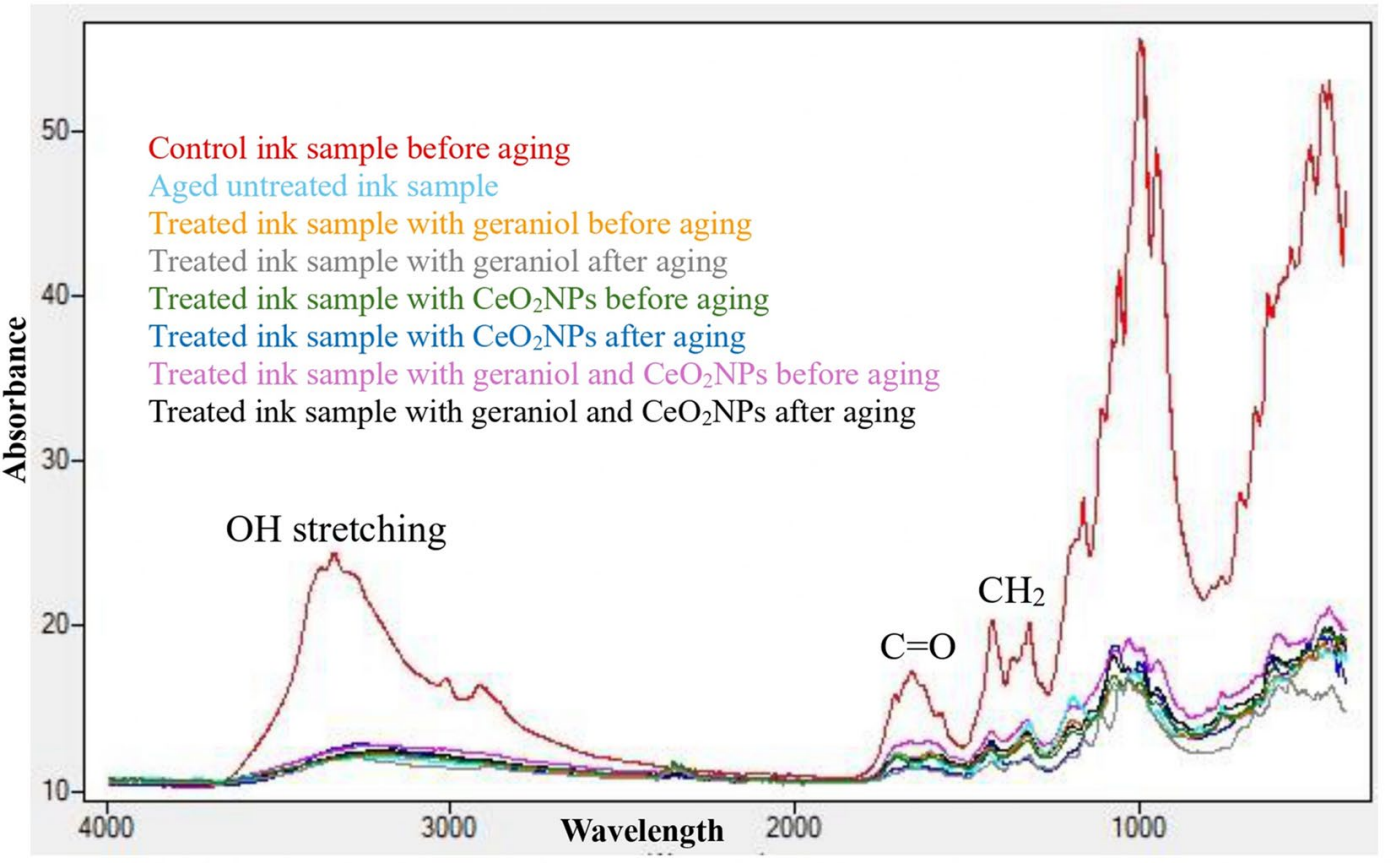
The ink sample, the treated paper samples with geraniol, CeO_2_NPs, and a geraniol-CeO_2_NPs mixture, both before and after artificial aging.

The application of FTIR spectroscopy to analyze the binding medium mixed with inks in Ottoman-era paper manuscript revealed a clear match with the standards of gum Arabic, with characteristic peaks observed at 3295 cm^− 1^ for OH stretching, 2924 cm^− 1^ for CH stretching, and C-O stretching at 1372 cm^− 1^. These features indicate that Arabic gum was used as the binding medium in the manuscript^27^ (Fig. 14).

**Fig. 15:**
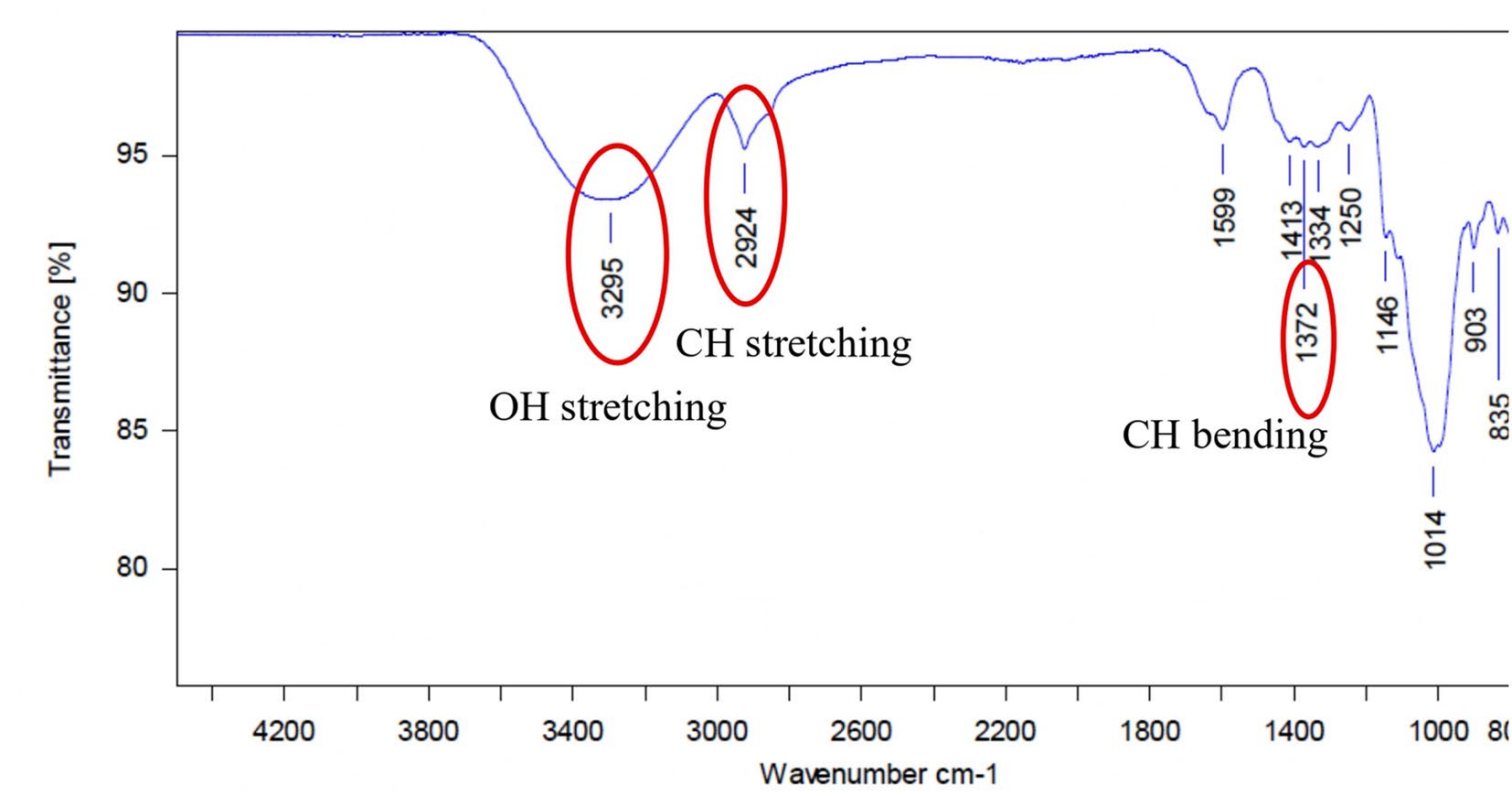
FTIR analysis of gum Arabic as a binder with inks in the paper manuscript.

### Conservation of the degrading Ottoman-era Islamic paper manuscript

#### Object description

**Fig. 16:**
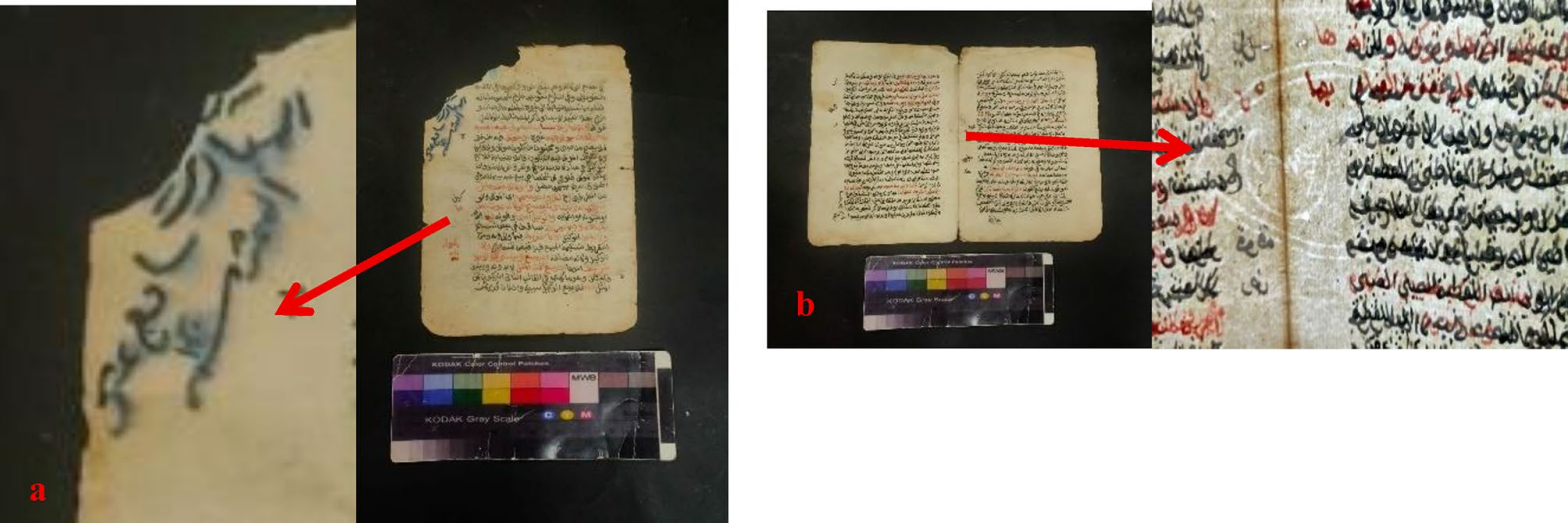
(**a**) the paper manuscript with a date from 17 Muharram 1030 AH, and (**b**) the watermark characteristic of the western pap.

The Ottoman-era paper manuscript (17 Muharram 1030 AH/1620 AD) is part of a private collection, highlighting exemplary Naskhi script. The manuscript is written on high-quality Western paper featuring a distinctive watermark linked to the esteemed Sampson factory in England. Measuring 27 cm in height and 16 cm in width, the manuscript consists of a single booklet adorned with red and black ink, accompanied by careful marginal notes. The manuscript’s structure includes three bifolios and a single sheet, with one page unfortunately missing. Its contents focus on religious law, offering insightful discussions on relevant rulings and principles. This manuscript thus provides a valuable look into the intellectual and spiritual heritage of its time (Fig. 16).

### Conservation stage

#### Fungal inhibition

**Fig. 17:**
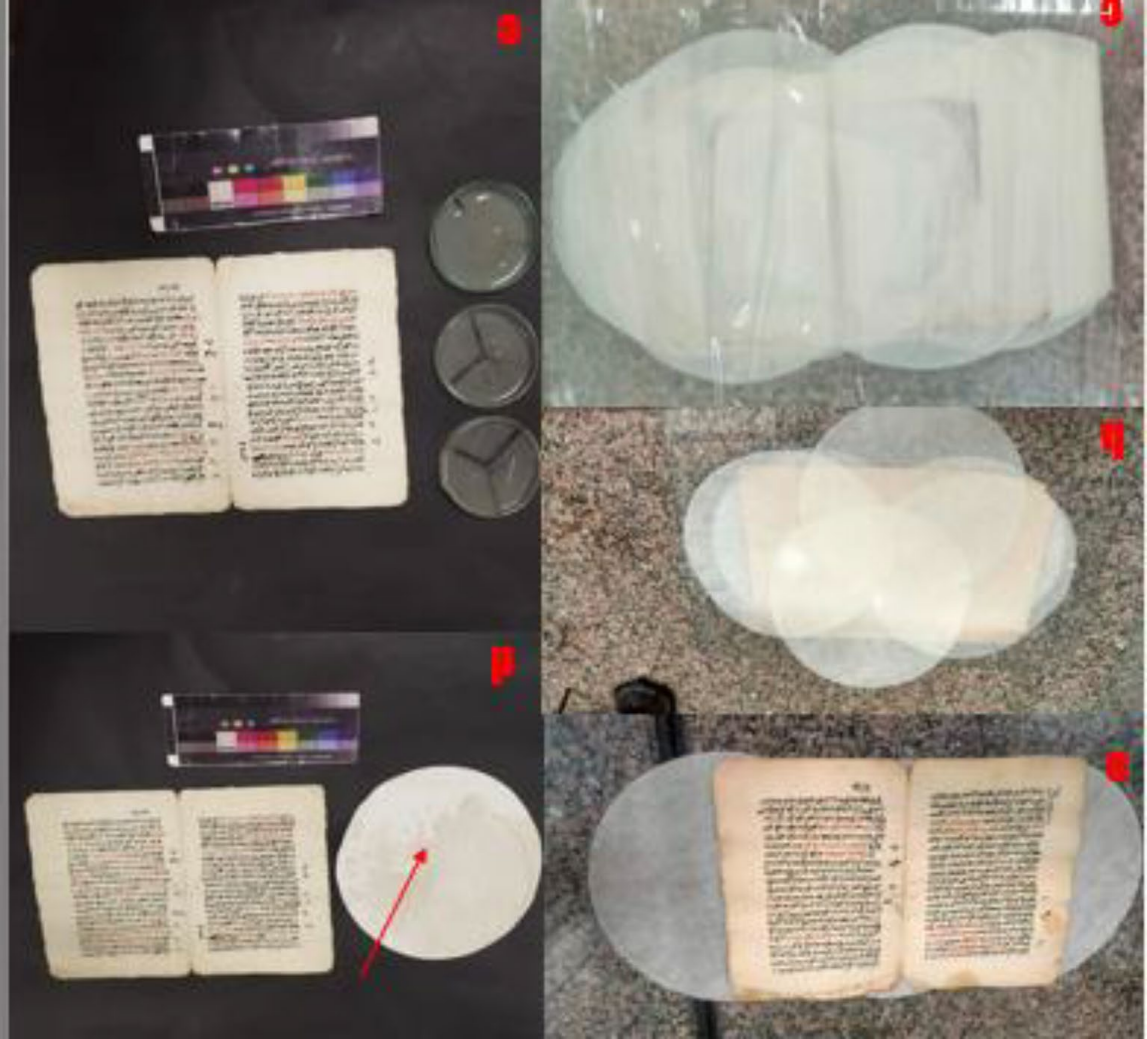
treatment of the paper manuscript with a geraniol-loaded paper poultice, (**a**) placement of the manuscript between paper layers, (**c**) closing the manuscript from all sides, (**d**) poultice after treatment, showing absorbed dirt and stains, (**e**) petri dishes confirming inhibition of fungal growth after treatment.

The poultice consisted of Whatman paper sheets soaked in geraniol at a concentration of 136.7 µL/mL with DMSO, and the optimal inhibitory concentration for fungal growth was applied to the paper samples. This treatment proved effective in reducing oxidation effects on the samples. After a 24-h application and removal of the poultice, a yellow discoloration and dirt were observed on the poultice material. This result confirms the poultice’s ability to draw dust from the surface without transferring or damaging the ink, thus maintaining its integrity during the process. Subsequently, samples from the manuscript were taken after fungal inhibition and cultured on PDA medium in Petri dishes. These were incubated at room temperature (25 °C ± 5 °C) for 7 days to verify the absence of fungal growth. The plates showed no growth of fungal mycelium, confirming the successful inhibition of fungal growth on the manuscript’s paper surface (Fig. 17).

### Dry cleaning and consolidation

The paper manuscript was cleaned using various mechanical tools, including soft brushes and acid-free erasers, applied with gentle circular motions. A scalpel was used to remove insect residue and any remaining solid dirt from the surface. After mechanical cleaning, the surface appeared significantly lighter, and all residues were completely removed, all without damaging the paper or its carbon and vermilion inks, and without the need for any chemical treatments.

Following the dry cleaning treatment, a thorough colorimetric measurement revealed a significant reduction in surface contaminants, resulting in a notable chromatic shift of 9.62. The decrease in the a* value from 5.74 to−3.58 indicates a substantial reduction in redness, due to the removal of chromatic impurities. In contrast, the subsequent consolidation treatment with 2% Klucel G loaded onto 0.25% ZnONPs induced a minimal color change of 1.52, suggesting that the treatment did not significantly impact the colorimetric properties of the paper manuscript. This outcome underscores the treatment’s efficacy in preserving the manuscript’s original appearance while maintaining its chromatic integrity. The consolidation treatment not only consolidated the surface of the manuscript but also effectively addressed its acidity.

X-ray diffraction analysis indicated that the cellulose possessed a crystallinity index of 75.2%. Consequently, the paper underwent a consolidation treatment using a 2% Klucel G solution of 0.25% ZnONPs in ethyl alcohol, which was carefully applied to both sides of the manuscript with a soft brush^28^. The manuscript was then allowed to dry completely to ensure stable and effective consolidation (Fig. 17; Table 2). The pH level of the manuscript, which was 5.8 before treatment, increased to 7.5 after treatment, indicating a significant reduction in acidity and a more stable chemical environment for the manuscript’s preservation.

**Fig. 18:**
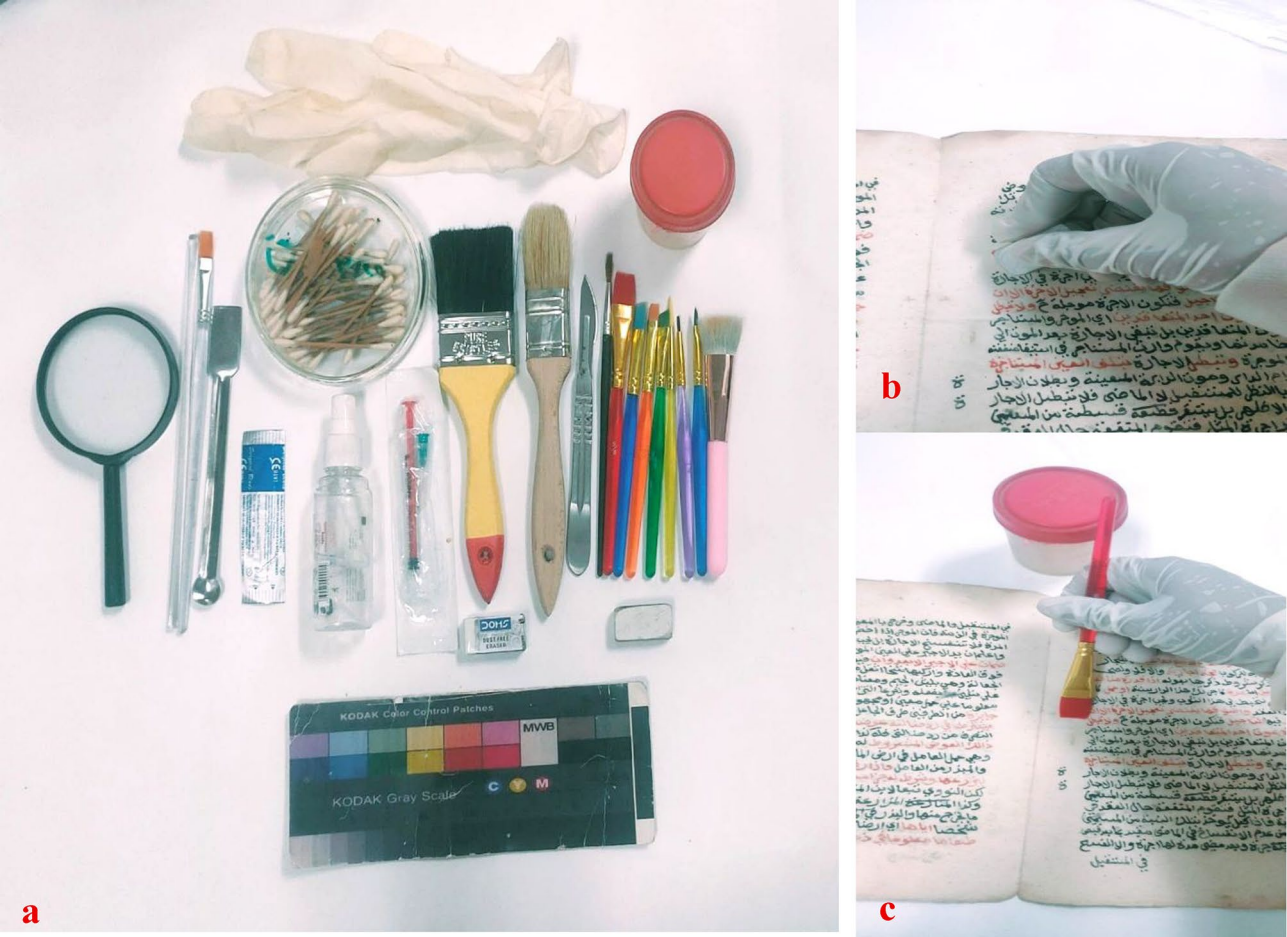
(**a**) The tools used in dry cleaning, (**b**) acid-free erasers, and (**c**) the application of a consolidation material composed of a 2% concentration loaded with 0.25% of ZnONPs.

**Table 2:** Color change measurements of the paper manuscript before treatment, after the cleaning process, and after the consolidation process.

Samples	(L)	(a)	(b)	$\Delta$E
Applied sample before treatment	76.00	5.74	14.86	-
Applied sample after cleaning	78.27	-3.58	15.68	9.62
Applied sample after consolidation	76.77	-3.53	15.41	1.52

### Compensation for losses and restoration of tears

**Fig. 19:**
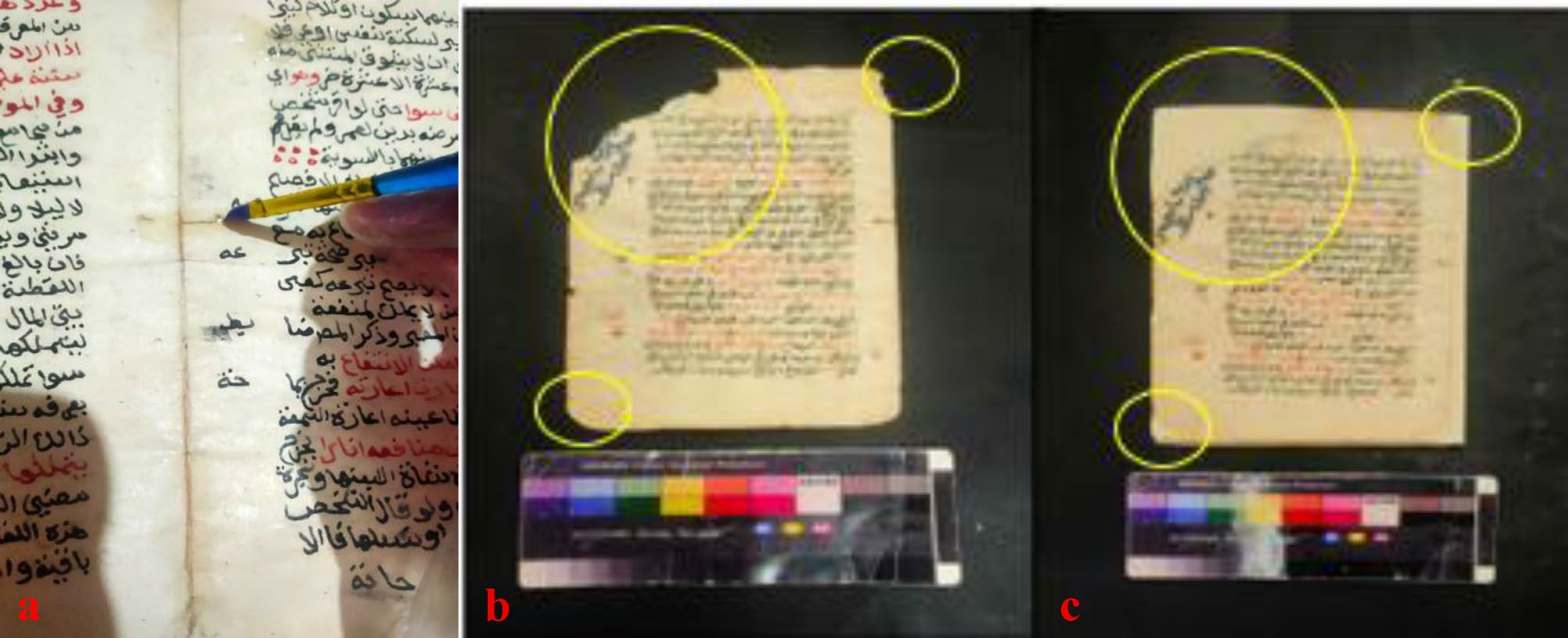
(**a**) treatment of tears using dyed tissue paper and adhesion with a 5% concentration of Klucel G, followed by infilling of losses (**b**) before (**c**) and after the infilling process.

Tears in the manuscript were repaired using a 5% Klucel G solution in ethyl alcohol, applied with a brush on the two sides^29^. Losses were restored using cotton paper pulp, carefully dyed with tea extract to match the original paper’s color and texture, developed at the wood pulp lab of the Egyptian National Library and Archives in Egypt. The repair process also involved the use of 5% Klucel G in ethyl alcohol for mending tears, supplemented by dyed Japanese tissue paper when necessary to ensure a durable and aesthetically pleasing repair (Fig. 19).

**Packaging**.

Acid-free paper, 300 g, with a pH of 8, was used in the process of packaging the paper manuscript after completing all its restoration processes, to make a box made of paper to protect the paper manuscript inside it. Two sheets were cut in the shape of a rectangle larger than the dimensions of the paper manuscript, 28 cm and 18 cm, and the height of the box was 1 cm in each direction. Then they were placed in a crossed manner and were adhered using Klucel G 5% in the area of intersection of the two sheets. Then all four parts of the box were folded to close the shape on the paper manuscript inside it, giving us the shape of a box to protect the paper manuscript. To provide the antioxidant and antifungal properties to the paper box intended for conserving the restored ancient manuscript, geraniol was utilized, and 123.8 mL of the prepared compound was introduced at a concentration of 136.7 μL/mL with DMSO in a beaker, which was placed in the lower compartment of a desiccator. The paper box was positioned in the upper compartment, and the desiccator was sealed to ensure a controlled environment. The treatment was maintained for a period of 14 days, allowing for the complete vaporization of geraniol for durable antifungal and antioxidant properties to the paper box (Figs. 19 and 21).

**Fig. 20:**
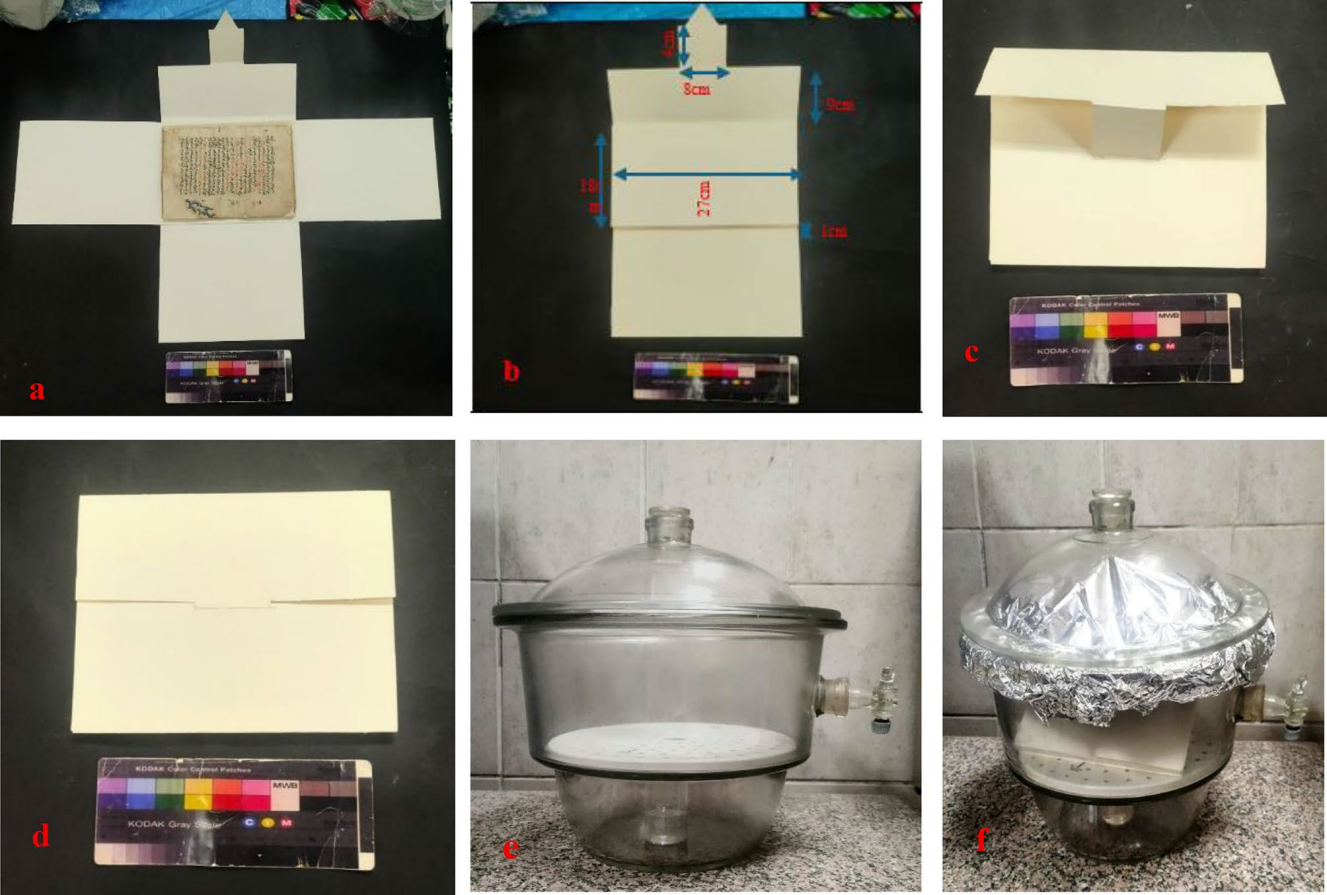
(**a**) Packaging of the paper manuscript involved placing it between two acid-free paper sheets, (**b**) adhered with a 5% Klucel, (**c, d**) the box was then closed to contain and protect the paper manuscript, using vapors of geraniol inside a desiccator, (**f**) vaporization of the paper box.

**Fig. 21:**
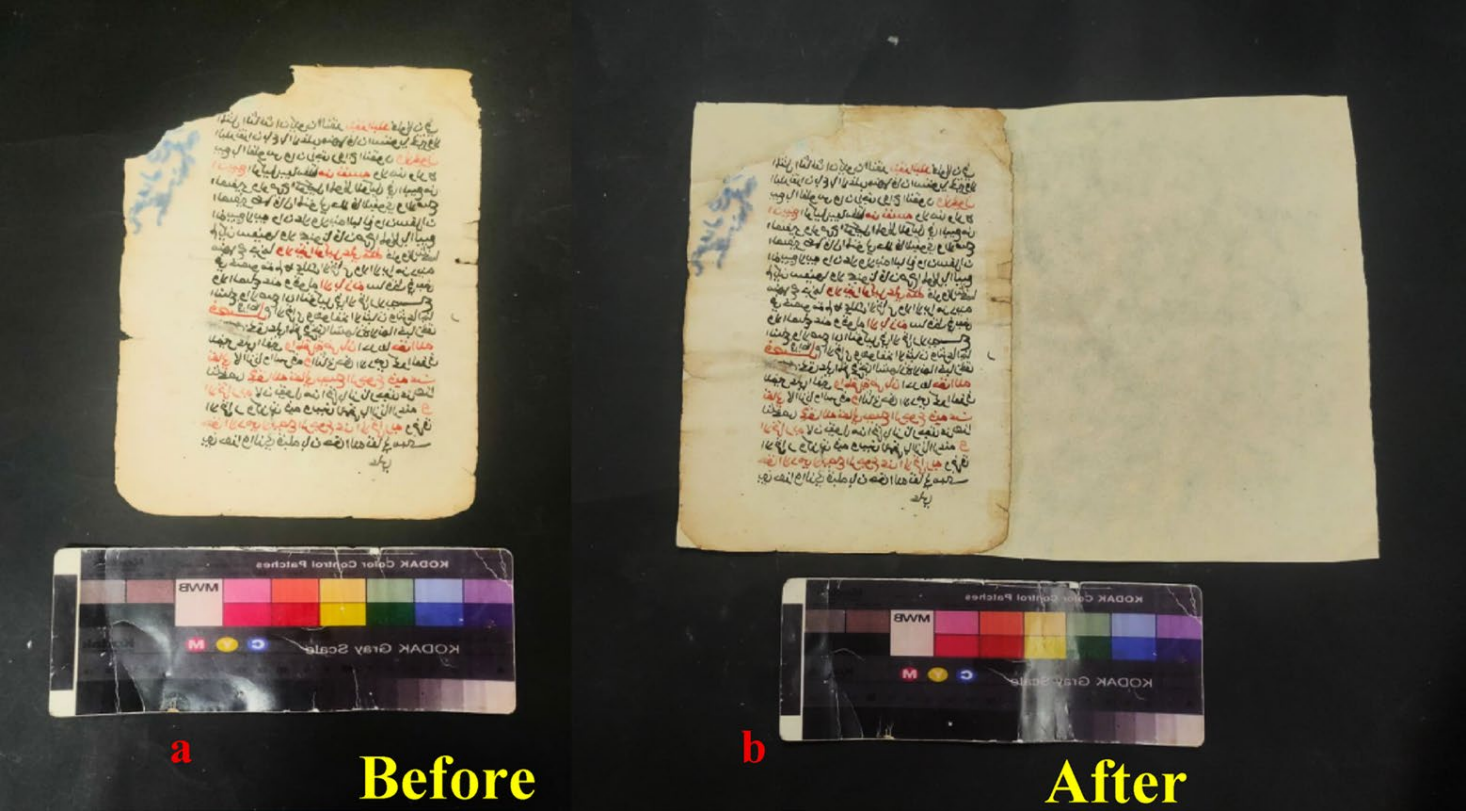
The paper manuscript (**a**) before (**b**) and after the completion of all restoration processes.

## Discussion

Evaluating the effectiveness of restoration materials through thorough examinations and analyses is crucial for selecting the best options that do not alter the characteristics of manuscript papers and their contents, including ink. This study demonstrated that geraniol at a concentration of 136.7 µL/mL is an effective antioxidant against UV radiation and acts as an antifungal agent against *Aspergillus terreus* and *Aspergillus fumigatus*. The antifungal properties of geraniol may be related to changes in the permeability of cell membranes, walls, and DNA, which can affect gene expression, cellular respiration, and energy metabolism, ultimately leading to cell death, according to references^30,31^. Therefore, after taking swabs from the samples treated with geraniol or from the manuscript paper in the applied area, no fungal growth was observed for 54 days.

Notably, geraniol preserved the properties of paper samples and iron gall ink. When applied to the paper manuscript from the Ottoman era, the geraniol-treated paper showed successful results, effectively removing fungal growth, dirt, and dust while achieving its intended purpose. Because the poultice system is the interaction of two porous materials in hydraulic contact^32,33^, and this process depends on ambient factors such as humidity and temperature, material qualities like composition, pore size distribution, porosity, and the presence and distribution of liquid and vapor phases within the substrates^34^ influence the operation. As a result, all contamination on the paper migrated from the surface into the paper poultice, resulting in a clearer surface compared to before treatment. This was confirmed by scanning electron microscopy, which showed the disappearance of fungal hyphae after treatment. Additionally, the surface roughness of the samples decreased from 2470.36 nm in the infected paper to 180.37 nm in the treated sample.

As the references indicate, CeO_2_NPs decreased fungal spore germination on solid surfaces. CeO_2_ inhibited fungal growth, especially under UV irradiation, and induced reactive oxygen species (ROS) formation, thereby altering cellular responses, so it functioned as an antioxidant and antifungal agent^35,36^. It inhibited fungal growth individually at a concentration of 18.11 µg/mL and when combined with geraniol. Despite this, cerium oxide nanoparticles did not achieve the same level of success as a multifunctional material like geraniol, partly because they caused a slight color change in the samples, which increased when mixed with geraniol. Furthermore, it led to brittleness and weakness in the samples.

The FTIR analysis showed the presence of a carbonyl group (C = O), indicating oxidation^26^, which disappeared after treatment with geraniol, whether before or after oxidation. This suggests that geraniol has an antioxidant effect. When using cerium oxide nanoparticles alone or combined with geraniol, peaks appeared in the C = O region, indicating their susceptibility to oxidation from artificial aging using UV radiation. However, geraniol retained its properties after UV aging without affecting the properties of the paper samples with iron gall ink, demonstrating its effectiveness as a multifunctional material.

Geraniol’s antioxidant and antimicrobial properties help preserve paper and iron gall ink by reducing oxidative degradation and inhibiting microbial growth. This study of geraniol for paper conservation offers a novel, eco-friendly approach compared to traditional methods, enhancing durability without altering the manuscript’s appearance. Potential limitations of the study include the long-term stability of CeO_2_NPs and the scalability of the treatment for large-scale applications. Further research is needed on more aging effects and applicability to CeO_2_NPs, adding another material to it, and improving its properties.

## Conclusion

Applying a geraniol-loaded Whatman paper poultice without CeO2NPs provides a highly effective and innovative method for conserving paper-based cultural artifacts. This plant-derived compound (Geraniol) exhibits a wide range of bioactivities, including antifungal effects against *Aspergillus fumigatus* and *Aspergillus terreus*, as reported in the paper manuscript. The treatment’s ability to reduce oxidation damage by UV and remove surface contaminants like dust and fungal mycelia was confirmed through detailed surface imaging using SEM and AFM. Additionally, the treatment did not alter the pH values of the samples, thereby preserving their chemical stability. The successful application of this method on an Ottoman-era paper manuscript highlights its potential for preserving valuable cultural heritage objects while maintaining their original properties and structural integrity. This approach also supports Egypt’s Vision 2030 for sustainability by emphasizing the use of eco-friendly and safe materials in all fields.

## Data Availability

All data generated and analyzed during the current study were available in the manuscript.
