# Correction: An eco-friendly evaluation of geraniol and CeO_2_NPs paper poultices for multifunctional paper manuscript conservation

**DOI:** 10.1038/s41598-026-57380-x

**Published:** 2026-06-11

**Authors:** Salwa M. A. Mahmoud, Maisa M. A. Mansour, Maha A. Ali, Mohamed Z. M. Salem

**Affiliations:** 1 Conservation Department, Faculty of Archaeology, Cairo University, Giza, 12613 Egypt; 2 Forestry and Wood Technology Department, Faculty of Agriculture (El- Shatby), Alexandria University, Alexandria, 21545 Egypt

Correction to: *Scientific Reports* 10.1038/s41598-026-49698-3, published online 06 May 2026

The original version of this Article contained an error in Figure 5, where the image in panel C was inverted. The original Figure 5 and accompanying legend appear below.

**Fig. 5:**
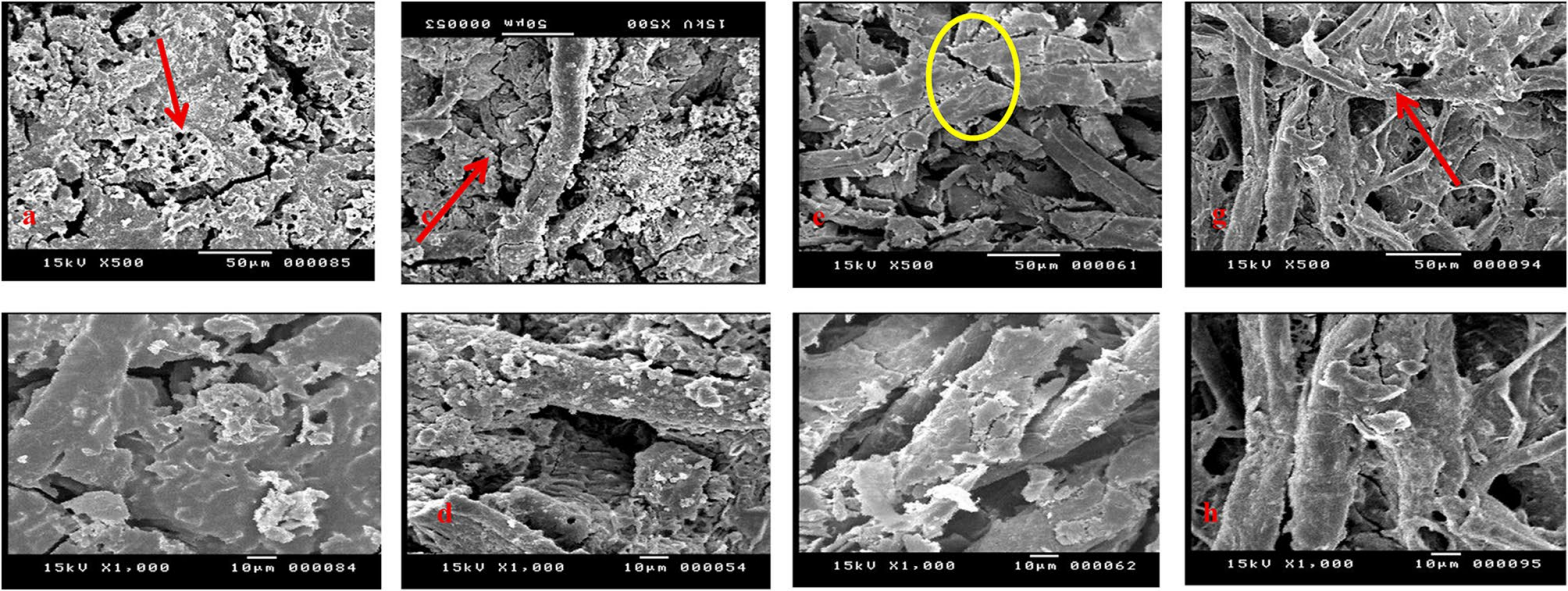
The paper samples with iron gall ink pre- and post-aging: (**a–b**) control, (**c–d**) geraniol-treated, (**e–f**) CeO_2_NPs-treated, and (**g–h**) treated with both, showcasing treatment effects.

The original Article has been corrected.

